# In the Search of Fundamental Inner Bond Strength of Solid Elements

**DOI:** 10.1155/2014/347360

**Published:** 2014-03-02

**Authors:** Maziar Sahba Yaghmaee, Reza Riahifar

**Affiliations:** Ceramic Department, Materials and Energy Research Center, P.O. Box 31787-316, Karaj, Iran

## Abstract

In order to understand the physics behind the surface properties and nano-scale phenomena, we are motivated first to investigate the inner bond strengths as well as the effect of number of neighboring atoms and their relative distance in addition to space positions (crystallography). Therefore, in order to study the effect of the nature of metallic bond on their physico-chemical properties, we first tried to investigate and introduce a mathematical model for transforming the bulk molar cohesion energy into microscopic bond strengths between atoms. Then an algorithm for estimating the nature of bond type including the materials properties and lattice scale “cutoff” has been proposed. This leads to a new fundamental energy scale free from the crystallography and number of atoms. The results of our model in case of fundamental energy scale of metals not only perfectly describe the inter relation between binding and melting phenomena but also adequately reproduce the bond strength for different bond types with respect to other estimations reported in literatures. The generalized algorithm and calculation methodology introduced here by us are suggested to be used for developing energy scale of bulk crystal materials to explain or predict any particular materials properties related to bond strengths of metallic elements.

## 1. Introduction

Binding strength inside the material which is mainly expressed by cohesion energy is classified as a bulk thermodynamics property. Such energy scale is believed to rule most of the physicochemical and mechanical properties of material [1, 2]. However, even the fundamental researches at nanoscale and surface science (as the new area of materials science) have been interrelated strongly with the cohesion phenomenon [3, 4]. Therefore, any generalized hypothesis at bulk, nano-scale, or even surface of materials should have been granted on the well developed cohesive scale of bulk materials.

In researches including bulk metal properties not only the macroscopic properties such as cohesion energy but also the number of neighboring atoms and crystallography of material along with the effective length of interaction energies between atoms play dominant roles in fundamental theories [5–7].

Moreover, in recent years bond energy calculation has been applied in estimation of surface properties through (atomic scale) broken bond model; thus the bond strength should be known between different neighboring atoms. In latest works of Fu et al. in [8–10] these estimations have been mostly supported by complicated quantum chemistry information through empirical electron theory where the bond energy of neighboring atoms was evaluated from the value of bonding capability of covalent electron, screen factor upon the core electron, bond length, and the number of covalent electron pair. Obviously, lack of a simple and more engineering based procedure has been realized which leads to an inquiry for developing a generalized simple method.

Therefore, we are motivated to closely study the structural effect of different crystals on their cohesion energy and evaluate a structural free energy scale which defines the materials properties. In this report, we study the effect of crystallography and number of atomic neighbors on the cohesive energy in order to define a fundamental energy scale of material. This information not only enables us to evaluate the real interatomic bond strength inside the material but also can be used as a grand energy scale to investigate other fundamental properties of elements. This knowledge gives us a tool to evaluate the strength energy from a particular atom inside the material. Also we develop free software using which anyone can compute the bond strength scale at different distances as function of number of atoms and their relative distances for any crystal materials by knowing some simple input data without any adjusting parameters.

## 2. Theory

Most problems in solid state science require detailed study of energies along with the atomistic structural information inside the material. A detail study of crystallography effect and its related features on inner cohesive strength of pure metals needs a fundamental model which not only evolves macroscopic cohesion properties but also includes the number of atoms (those which are affected by cohesive force of an arbitrary inner atom) and their relative distances inside the materials. In order to fulfill such conditions we need to develop a model for distribution of cohesion energy between the (effected) atoms as a function of their number and relative distances from an arbitrary inner atom.

### 2.1. Modeling the Solid Materials

In most of theoretical investigation in materials science dealing with thermodynamics description of material, the dimension of cohesion energy is described as a molar quantity. Therefore, theoretically by applying that amount of energy to the material we can dissociate one mole of material into its atoms [1]. In order to investigate the amount of any particular bond strength we need to find an algorithm for transferring macroscopic (molar) cohesion energy into atomic (inner) bond strength scale.

**Figure 1:**
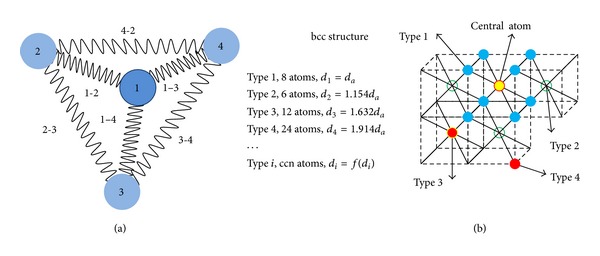
Schematic representation for (a) number of bonds related to number of atoms and (b) position of equal distance atomic groups and their distances from an arbitrary (inner) atoms in bcc lattice.

Regarding Figure 1(a), as long as we consider all bonds between any two particular atoms inside the material, then, the total cohesive strength of *N* atoms leads to *N*(*N* − 1) of total bonds. However, as we count each bond twice, the total number of bonds inside material with *N* atoms will be

(1) $$\begin{array}{c} \frac{1}{2}\left(N\left(N-1\right)\right)=n_{b}, \end{array}$$

where *N* is the total number of atoms and *n*_*b*_ is the number of bonds in material.

Therefore, during the transformation of molar cohesive energy of material into the atomic bond strength scale, we have to apply a 1/2 coefficient in order to avoid counting of multiple bonds between two particular atoms. Considering a simple case of Figure 1(a) where we have only 1 type of inter atomic cohesive strength of *E*(*ij*) between each pair of atoms of *i* and *j*, then regarding (1) we can write

(2) ENtotalE(ij)=nb⟹ENtotalN=12(N−1)E(ij)⟹E1↔N−1total=12(N−1)E(ij),

where $E_{N}^{\mathrm{total}}$ is the cohesive strength of *N* atoms, $E_{1↔N-1}^{\mathrm{total}}$ is the cohesive strength between 1 inner and all neighboring atoms, and *N* − 1 is the number of neighbors (denoted by ccn (cohesion coordination number (in case of first ccn we reach the value of classical *CN*))).

However, in reality for an arbitrary inner atom we have more than one type of cohesive strength as Figure 1(b) shows. Therefore, if we consider a homogeneous cohesive energy distribution (in all directions of crystal cell) among each group of atoms with equal distances from an arbitrary inner atoms (far enough from the surfaces), we can write

(3) $$\begin{array}{c} E_{\text{inner}}^{\text{total}}=E_{\text{inner}}^{}\left(1\right)+E_{\text{inner}}^{}\left(2\right)+\cdots =\sum_{i}E_{\text{inner}}^{}\left(i\right), \end{array}$$

where *E*_inner_(*i*) is the total cohesion energy of *i*th group of atoms in [kJ/mol], $E_{\text{inner}}^{\text{total}}$ is the total inner cohesion energy of material in [kJ/mol], and *i* as the atomic group that represents the bond type.

We also suppose that the distribution of cohesive energy is homogenous between all atoms in each group ccn; therefore, regarding the coefficient of atomic bond scale in (1), the total cohesive energy in (3) can be rewritten as

**Figure 2:**
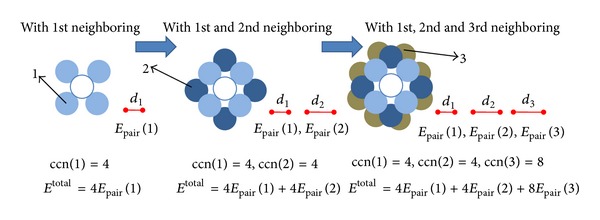
Simple schematic to illustrate how the number of atoms and their relative distances evolve in philosophy behind (4).

(4) Einnertotal=12ccn(1)Epair(1)+12ccn(2)Epair(2)+⋯=12∑iccn(i)Epair(i),

where *E*_pair_(*i*) is the average cohesive energy (bond strength) in *i*th atomic group between each of the atom in that group and the particular inner atom from which we count the distances in [kJ/mol] or [kJ/mol no.] (see also Figure 2).

However, as we fundamentally subtract out the effect of number of neighbors by introducing the ccn(*i*) in (4), the effect of distance on the cohesive strength between all atoms and the arbitrary inner origin atom should be considered inside the *E*_pair_(*i*) function. Such contribution attributes through soft-sphere model [7] to most of the pair potentials theories [11–14], where the parameters with energy dimensions can be extracted out from the distance or lattice parameters terms. In aforementioned models, all pair potentials (energy terms) are supposed to be constant as a material property. Therefore, developing further (4) leads us to define a distance related dimensionless function *f*(*d*_*i*_) along with a fundamental energy scale *E* ^av^, as

(5) $$\begin{array}{c} E_{\text{pair}}^{}\left(i\right)=f\left(d_{i}\right)E^{\text{av}}, \end{array}$$

where *d*_*i*_ is the relative atomic distance of *i*th group of atoms from an arbitrary atom in (nm) and if *i* : max⁡. then *d*_*i*_ = *d*_cutoff_ representing the maximum distance for effectiveness of bond strength.

Further on, by introducing (5) into (4), the total cohesion energy of material can be written as

(6) $$\begin{array}{c} E_{\text{inner}}^{\text{total}}=\frac{1}{2}\sum_{i}\text{ccn}\left(i\right)f\left(d_{i}\right)E^{\text{av}}=E^{\text{av}}\frac{\sum_{i}\text{ccn}\left(i\right)f\left(d_{i}\right)}{2}. \end{array}$$

### 2.2. Nature of Distance Function *f*(*d*_*i*_)

In (5) *f*(*d*_*i*_) is not a simple length related (metric) function, but it represents the fundamental nature (physics) of bonds among the atoms of a particular solid at different distances with respect to an arbitrary inner atom. By applying the soft-sphere model [7] and considering all potential function theories like embedded-atom model [12] EAM, Finnis-Sinclair model [11, 13] FS, or even the simple Lenard-Jones potential [15] LG, we consider an inverse power potential form for physics of bond strength. Therefore, such hypothesis would be a quite adequate approximation for defining the distance related cohesion energy term inside the crystal metals. Thus, we can write

(7) $$\begin{array}{c} f\left(d_{i}\right)\propto \frac{1}{d_{i}^{n}},\;\text{where}\;n=1,2,\ldots , \end{array}$$

where *n* defines the nature of particular binding inside material in a given crystal structure and is called power of potential function.

The *f*(*d*_*i*_) function should include atomic diameter *d*_*a*_ as a material property and the crystallographic parameter which is the geometrical position of each atom from an arbitrary inner atom. Therefore, we can write

(8) $$\begin{array}{c} f\left(d_{i}\right)\;\text{also}\propto \left(d_{a},\text{geometry}\right). \end{array}$$

If we measure the distances of each atom from an arbitrary inner atom (equicentral spheres), the first coordination distance *d*_1_ can be defined at contact condition of two neighboring atoms *d*_*a*_. Also *f*(*d*_*i*_) should remain dimensionless; therefore, by summarizing the aforementioned three constraints on *f*(*d*_*i*_) we can conclude that

(9)  (i)(7)⟼f(di)∝1din (ii)  also  if  i=1⟼d1=da           ⟹f(di)=dandin. (iii)(5)⟼f(di)  is  [nm/nm]⟼f(di)∝dadi,

As it can be seen from (9), value of *f*(*d*_*i*_) always represents a relative distance feature of cohesion effectiveness respect to the first contact coordination neighbor. Thus, if we have only the first neighboring coordination atoms, then each atom senses a maximum cohesive strength as *f*(*d*_1_) = 1, while by increasing the number of atoms and being placed far from the first coordination atoms (ccn = 1), the relative potential energy exponentially decreases [11–13, 15] by power *n* as (7) shows it.

Therefore, if we would be able to construct the mathematical algorithms of all geometrical positions in fcc, bcc, and hcp crystallographic structures, then we would be able to extract out the *d*_*a*_ from the *f*(*d*_*i*_) function by applying the mathematical series for each atomic group distances by *d*_*i*_ = *d*_*a*_ *g*_crystal_(*i*). Thus, (9) can be simplified to

(10) $$\begin{array}{c} ⟹f\left(d_{i}\right)=\frac{d_{a}^{n}}{{\left(d_{a}g_{\text{crystal}}^{}\left(i\right)\right)}^{n}}=\frac{1}{g_{\text{crystal}}^{n}\left(i\right)}. \end{array}$$

and by substituting the result of (9) into (6) we can write

(11) Einnertotal=Eav∑iccn(i)f(di)2=Eav∑iccn(i)/gcrystaln(i)2⟹Einnertotal=EavF(distance  &  geometry),

where *F* is symbolizing a function which includes ccn(*i*) and *g*_crystal_(*i*) values.

Equation (11) shows that macroscopic cohesion energy $E_{\text{inner}}^{\text{total}}$ can be expressed as multiplication of a fundamental energy scale *E* ^av^ and function *F* which is a complicated function of number of atoms and their relative distances. Obviously function *F* should converge to a value in order to express the dependency of $E_{\text{inner}}^{\text{total}}$ from *E* ^av^ by the effect of atomic group distances and geometry of crystal. Thus, by knowing the total $E_{\text{inner}}^{\text{total}}$ as an overall thermodynamics quantity [1], the mathematical series which describe the equilibrium atomic distances (based on crystal structures) from a particular inner atom, and the number of neighboring atoms in each distances, along with knowing the value of *n* (power of potential function: nature of interaction between the atoms), then we are able to subtract out the influences of distance, geometry, and number of atoms. Therefore, this leads to a fundamental energy scale *E* ^av^ which is free from any crystal structure information.

Before any analytical investigation, we have to study the crystallography of different structures and evaluate the required mathematical series which represent the equal atomic distances *d*_*i*_ and number of neighboring atoms ccn(*i*).

### 2.3. Distance and Relative Number of Neighboring Atoms in fcc, bcc and hcp Lattices

There were already some attempts in literature to evaluate the effect of number of neighboring atoms and in some cases the effect of their relative atomic distances mostly for estimation of surface properties of pure metals in fcc, bcc, and hcp structures in [8, 9, 17, 18] and [10, 19, 20], respectively. The value of bond energy in this literature is originated from either empirical electron theory, dangling bond analysis methods, atomic potential simulation, or density function theory which mostly are based on parameters such as covalent electron pairs, bond length, number of electron, contribution of couple effect between spin and orbit, bond capability, screen effect, electron density, and much other quantum chemistry information.

In their reports Zhang et al. in [17] considered only the effect of 1st nearest neighbors for fcc metals, and Fu et al. in [8, 9] used up to 3rd nearest neighbors for fcc and bcc, respectively, and in [10] used up to 7th nearest neighbors for hcp structures, while Wu et al. in [18, 20] considered up to 12 slabs for fcc and hcp surfaces, respectively, and Matysina in [19] considered up to 3rd nearest neighbors effect for hcp crystal structures.

Unfortunately neither the application of above literature resources nor their mathematical formulations can provide a generalized mathematical series for estimating the number of neighboring atoms and their relative distances. However, Sloane and Teo in [21] reported a magnificent but complicated mathematical formulation for theta series and magic numbers in closed packed clusters (see also Appendix A) which yet few applications are known in which; one exactly can use this information in materials science researches.

**Figure 3:**
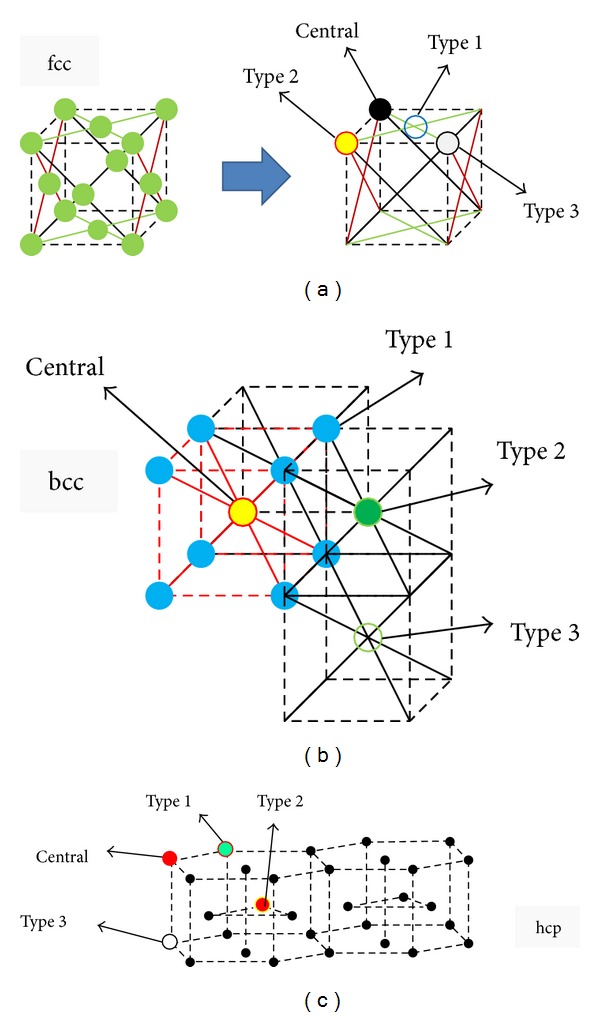
Schematic view of first three atomic groups and their relative distances from an arbitrary inner atom in (a) fcc, (b) bcc, and (c) hcp structures.

However, not only our different viewpoint of such series but also our free-software program (based on information appearing in Appendix A) enables every researcher to evaluate and apply the effect of up to 50th neighboring atomic groups and their relative distances from an arbitrary inner atom in fcc, bcc, or hcp structure. For a simple representation Figure 3 shows examples up to 3rd nearest neighbors in fcc, bcc, and hcp cells.

Regarding (10) and (11) mathematical series presented in Appendix A enables us to evaluate the values of *g*_crystal_^*n*^(*i*) and ccn(*i*) at given *i*th neighboring groups.  Table 1 shows the result of first 15 neighboring atoms and their relative distances in fcc, bcc, and hcp crystals. Appendix A shows the generalized mathematical series which is able to reproduce these numbers. In addition, developed free-software computer code is able to evaluate these calculations.

### 2.4. Flow Chart of Free Program and Its Algorithm

**Figure 4:**
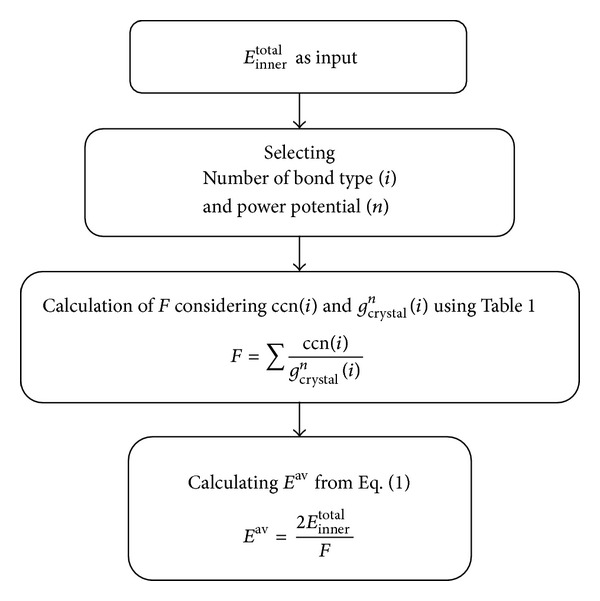
Algorithm of calculating *E* ^av^.

To facilitate calculation of *E* ^av^ based on our mathematical model, we have written a user friendly code named IBSE-Ver1 in visual basic. IBSE stands for *inner bond strength of solid elements*. In this software, first, appropriate value of $E_{\text{inner}}^{\text{total}}$ should be chosen. Second, number of bond types, *i*, and power in potential function *n* are determined by user. Finally, user chooses crystal structure of the element, bcc, fcc, or hcp. To easily select an element, a periodic table is shown after starting the program which enables user to choose an element from it. Initial input values including $E_{\text{inner}}^{\text{total}}$ and crystal structure are predefined in the software from [1, 16], or Table 6 for each element at 0 Kelvin. However, users can introduce their own values for $E_{\text{inner}}^{\text{total}}$ or crystal structure. Also users can change *n* and *i* to see the effect of these parameters on *E* ^av^. The algorithm of calculations is shown in Figure 4 as described completely in the text.

After clicking the calculate button, three kinds of information are shown as output of the software. First, the value of *E* ^av^ is shown in the text box depending on the value of input parameters ($E_{\text{inner}}^{\text{total}}$, crystal structure, *n*, *i*). Second, *E* ^av^ is depicted against number of selected bond types, *i*, in a graph. Third, there are buttons which by clicking them user can see some alternative information. More information about IBSE-Ver1 is available in the help of this software.

## 3. Results and Discussions

### 3.1. How to Select the Input Information

Regarding the aforementioned hypothesis in (11) in addition to $E_{\text{inner}}^{\text{total}}$ we need to know the maximum number of atomic distances *i* (cutoff length) and the nature of bonds *n* (power of potential function) inside that particular material. For a given material at a particular environment conditions while knowing the value of $E_{\text{inner}}^{\text{total}}$ at given temperature and pressure for a particular lattice structure (fcc, bcc, or hcp), Appendix A enables us to evaluate the number of neighboring atoms and their relative distances from an arbitrary inner atom. Obviously, this information can be achieved from our free-software program too. However, in order to estimate the value of *E* ^av^ we need to calculate the quantity of *F*. In other words, by knowing the value of *i* and *n* we are able to calculate the *F* = *f*(*i*, *n*) function which is needed to estimate the value of *E* ^av^.

One of the advantages of modeling the nature of bond strength by introducing the dimensionless function *f*(*d*_*i*_) in (9) was the extraction of materials properties such as atomic diameter (see also (10)). Therefore, function *F* = *f*(*i*, *n*) depends on the number of bond types *i*, which should be considered along with the number of atoms in each of these groups ccn(*i*). This pure crystallographic information is one of the outcomes in Appendix A.

For sake of simplicity we will classify all metallic elements into three main lattice groups (fcc, bcc, and hcp) and try to evaluate the quantity of *F* for each of these groups collectively. Therefore, we can study the mathematical behavior of *F* in each three main crystallographic structures as function of *i* and *n*.

**Figure 5:**
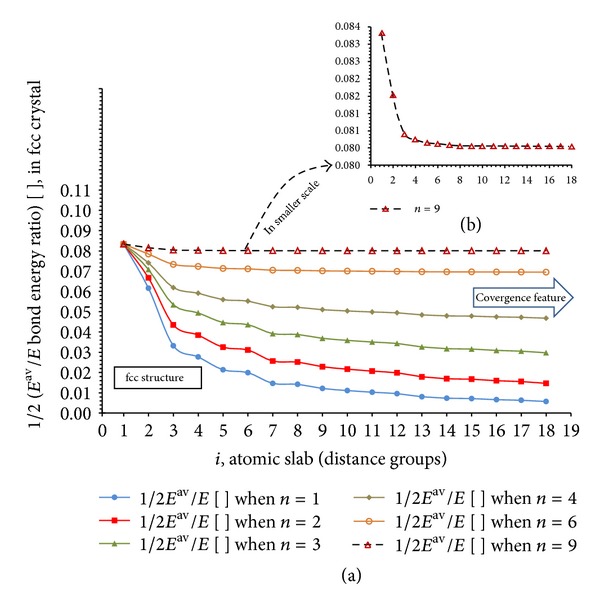
Calculated results of 1/*F*(*i*) as a function of *i* in different *n* values on the example of fcc crystal, (a) applying *n* = 1–4,6, 9 and (b) using *n* = 9 in smaller scale section.

Obviously, by increasing the value of *i* (increasing the effective bond length and number of bond types), the number of atoms sensing the chosen arbitrary atom from which these atomic groups are measured increases, but the bond energy that each atom experiences decreases too. Thus, there will be a length (cutoff) above which the addition of atomic groups is unrealistic. Therefore, regarding the geometrical and mathematical nature of *F* function we expect a convergence feature in each structure.  Figure 5 shows the results of such calculation for 1/*F*(*i*) on the example of fcc crystals up to 18 atomic slabs for different *n* values.

As it can be seen in Figure 5, by increasing the value of *n* the decreasing rate of cohesive force (bond strength) increases which lead to overall bigger value of 1/*F* function. In addition, by increasing the value of *i* above 8-9 a convergence feature is observable. Now that the convergence of *F* has been proved, we try to find an algorithm which enables us to estimate the optimum value of *i* (number of maximum slabs something similar to “cutoff”) and the nature of interaction which is represented by *n* in (10).

In classical literatures, value of *n* = 2,3, 4 attributes to ion-ion, ion-dipole, and dipole-dipole interactions behavior, respectively, whereas in latter pair potential models like LG values vary around 6 [15] while in FS model [11, 14] the square root power of distance effect for Ni, Cu, Rh, Ag, Ir, and Al was found to be 6 while for Pd and Pb it was fitted by 7 and for Pt and Au it became 8. But in the following we propose a simple algorithm using which value of *i* and *n* can be evaluated more adequately in a simple manner.

**Figure 6:**
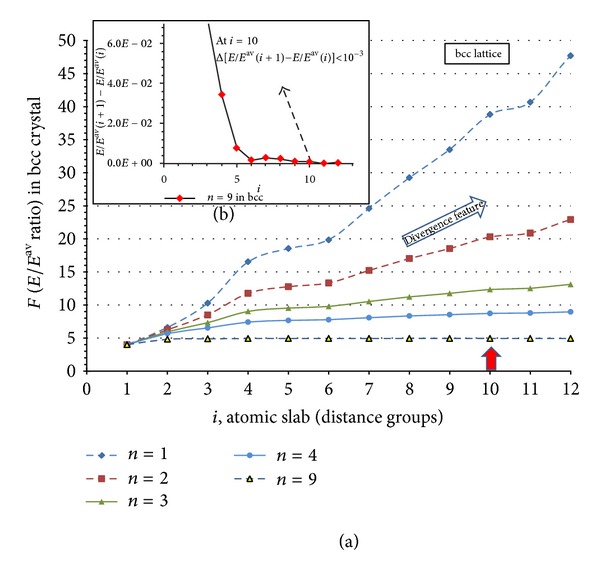
(a) Calculated results of *F* from (11) in bcc crystal for *i* = 1–12 and *n* = 1–4,9 and (b) the *F*(*i* + 1)/*F*(*i*) for *i* = 1–12 and *n* = 9.

Figure 6(a) shows the calculated results of *F* for different series *i* using different *n* values in case of bcc crystal. Obviously lower amount of *n* leads to divergent behavior of *F* function (see *n* = 1,2, 3, or 4 in Figure 6).

Proposed algorithm: regarding the behaviors of simple metals, we can overview the following simple three boundary conditions (two plus one) using which the acceptable values of *n* and *i* can be estimated.

- As *n* represents the nature of bonds between the atoms, its value should be between 6 and 9 in order to satisfy the basics quantum chemistry requirements; thus 6 ≤ *n* ≤ 9. Obviously, for a given problem *n* can be set differently.
- *i* represents the longest distance where an arbitrary atom can exerts its cohesive attraction force; therefore, it shows the so-called cutoff length of crystal. In simulation algorithms usually value of 2–2.5 a (a: lattice distance) is used [11]; therefore, regarding the values of Table 1, here we choose its corresponding value of *i* in different crystals. Therefore, *i* can either be estimated or the indirect experimental values of effective number of atomic layers can be transferred into the length scale.
- In addition to the above two conditions, we consider a limitation of 10^−3^ for the relative change of *F* = *f*(*i*, *n*) function in (11) while the values of *i*, and *n* vary. This condition shows the acceptable mathematical convergence limit for *F*; therefore, by varying *i* we have [*F*(*i* + 1) − *F*(*i*)] ≤ 10^−3^.

By applying the condition (a), while searching with condition (c) (Δ*F* ≤ 10^−3^; see Figure 6(b), e.g., of bcc lattice), the following atomic distances (slabs) have been found:

- in fcc by *n*: 6, 7, 8, and 9 at *i*: 23, 15, 11, and 8, respectively,
- in bcc by *n*: 6, 7, 8, and 9 at *i*: 17, 16, 11, and 10, respectively,
- in hcp by *n*: 6, 7, 8, and 9 at *i*: 19, 19, 15, and 13, respectively.

Therefore, considering the condition (b), using mathematical series in Appendix A in fcc, bcc, and hcp structures, respectively, the values of *i*: 11, 10, and 13 (with cutoff values of almost *≈*2.3 a, *≈*2.5 a and *≈*2.7 a) could satisfy all three conditions. These values of *i*: 11, 10, and 13 in fcc, bcc, and hcp belong to *n*: 8, 9, and 9, respectively, which will be used here for our calculation. Obviously, upon request applying similar algorithm using our free-software program a more reliable values can be generated for any particular condition.

### 3.2. Structural Free Cohesive Scale of Elements (Computation and Verification)

By factorizing out the crystallographic aspect of total cohesive energy regarding the classical or corrected cohesion scales [1] along with separating the effect of atomic distances in (11), the achieved energy scale *E* ^av^ represents the pure material characteristics of each particular metal. By analyzing the aforementioned hypothesis, in (11), one can say that the nature of four physical quantity $E_{\text{inner}}^{\text{total}}$, *E* ^av^, *g*_crystal_, and ccn can be expressed as follows

(12)  Einnertotal=f(T,da,material,crystal,…), gcrystaln(i)=f(n,crystal),           ⟹Eav≈f(material). ccn(i)=f(crystal),

Obviously, regarding the temperature dependency of relation (12) we know that by increasing the temperature from 0 Kelvin the lattice expansion along with increasing vacancy effects also should be considered. However, $E_{\text{inner}}^{\text{total}}$ as a macroscopic property at different temperature represents the real behavior of materials including the vacancies and lattice parameters; therefore, for the time being we neglect such contribution. The results of our calculation for *E* ^av^ at 0 K and at *T*_*m*_ for fcc, bcc, and hcp metal materials are presented in Table 2. As it can be seen some metals melt in different lattice crystal than they are usually in solid state at 0 K. Applying the attached free-software, one can produce more reliable values for structural free energy scale separately for any metallic element.* (The attached free-software in this paper is available at our website *(http://www.fcrgroup.org).* At our website under the Achievements main menu goes to Setups and Software submenu and find IBSE-Ver1 software. The terms of service/use (TOS) for attached free-software ARE the correct citation to this paper.).*

*E* ^av^ is mainly affected by the nature of atomic interaction between each pair of the inner atoms. Moreover, considering the potential function phenomenon developed in literature and our aforementioned hypothesis, we can say that *E* ^av^ = *f*(*n*). Thus, by rearrangement of (11) we can write

(13) $$\begin{array}{c} E^{\text{av}}=2E_{\text{inner}}^{\text{total}}\sum \frac{g_{\text{crystal}}^{n}\left(i\right)}{\text{ccn}\left(i\right)}⟼E\;\text{scale}. \end{array}$$

#### 3.2.1. Verification with Melting Points

The bulk melting phenomenon in crystalline materials takes place when the bindings of crystallographic cells lose its structure; thus the best verification properties for cohesive energy scale are believed to be the melting point [1]. The value of cohesion energy has been defined in classical literature as a negative value of sublimation enthalpy while in the past decades there have been several attempts for correcting these values in order to fit the melting point more adequately [1, 2].

**Figure 7:**
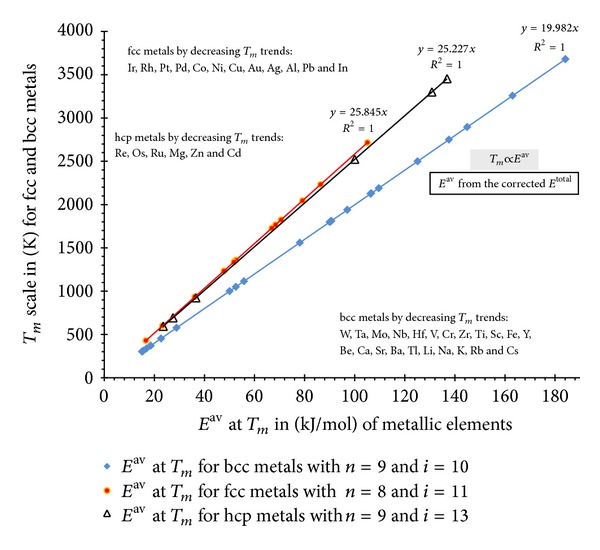
Verification of structural free cohesive scale with their melting points for fcc, bcc, and hcp crystals.

However, regarding the logic mentioned in (13) we expect that *E* ^av^ should verify more adequately the main physicochemical properties of materials such as melting with respect to classical cohesive energy scale. In the following this verification for three different main crystallographic forms has been shown (in Figure 7).

As our calculation results show, the correlation in three different crystallographic forms is perfectly represented by *E* ^av^ considering the boundary conditions of *i* and *n*. Therefore, we may introduce the *E* ^av^ concept as a simple structural free cohesive energy scale for pure crystal elements as a fundamental correlation scale regarding the physic-chemical properties of metals.

#### 3.2.2. Verification with Bond Strength for Different Neighboring Atoms

Applying the aforementioned computational algorithm in (11) and considering the relative atomic group distances in Table 1 along with the results of Appendix A, we are able to evaluate the bond strength inside the fcc, bcc, and hcp metals by *E*_pair_(*i*) in (4). *E*_pair_(*i*) simply expresses the bond strength at *i*th atomic groups (bond type).

The latest reported about the applications of bond strengths were for estimating the surface properties of pure metals in works of Fu et al. in [8–10] which the bond strengths were calculated using the empirical electron theory. Fu et al. in [8, 9] used up to 3rd nearest neighbors (bond types: A, B, and C) for fcc and bcc, respectively, which are compared to our calculation results in Tables 3 and 4. Obviously, not only the simplicity of our model but also the fact that neither the number of atomic neighbors nor the nature of bonds is a limitation here could give extra advantages for our method. In addition to proposed value of *i* and *n* in Table 2, a set of calculation with *i* = 3 (limitation in [8, 9] for fcc and bcc, resp.) and 2*n* for power of potential function shows more similarity to values in literatures (see Tables 3 and 4).

Fu et al. in [10] reported up to 7th nearest neighbor (bond types: A, B, C, D, E, F, and G) bonds strength for hcp structures which is compared also to our results in Table 5. As our hcp crystal has considered to be a perfect cell, between type A and B and type E and F the nearest neighbor value in [10] has been considered for the first and forth type bond strength. As a result, we evaluate up to 5th nearest neighbor values from [10] to compare with our results.

In case of hcp metallic crystals we also evaluated a set of calculation with *i* = 5 (limitation in [10] for hcp) while keeping 2*n* for power of potential function with respect to Table 2 which shows more similarity to values in [10]. These calculations illustrate the ability and flexibility of model and software for fitting and adjustment. As it can be seen in Tables 3–5, not only the tendency of rate of decreasing the bond strengths is similar, but also the absolute estimated values adequately fall in a comparable energy zone. Moreover, using our free-software and generalized algorithms for any metals in any crystal structure at a particular temperature, one can regenerate a new set of data.

## 4. Conclusions

A detailed investigation on the crystallography of fcc, bcc, and hcp lattices has been performed. This application of mathematical theta series gives us the information about the number of atoms and their relative equal distances from a central inner atom for up to 50th layers. Therefore, as long as the cutoff length (longest atomic distances from which an atom could sense the attraction of other one) is known, the maximum number of neighboring atoms and their distances from the central atom can be calculated.

Using these data, we introduced a model for transformations of bulk molar cohesion energy into inter atomic bond strengths inside the metallic elements. Obviously, in addition to number of neighboring atoms and their distances, the nature of bond type of particular material (as power in an inverse power potential) in given crystal structure should be known.

Therefore, an algorithm for estimating the value of neighboring atoms and distances from the central atom as function of crystal type and type of bond (nature of bond strength) has been proposed and for the sake of simplicity three collective results for all fcc, bcc, and hcp crystals have been evaluated and reported here.

The effect of number of neighboring atoms and their distance along with the physics of bond type inside the metallic elements has been investigated. This study leads to defining an energy scale free from crystallographic information. The aforementioned energy scale presented here is suggested to be used for scaling any fundamental properties of metallic elements which are interrelated to their inner cohesive feature.

We developed a free-software (IBSE-Ver1) which enables us to evaluate the number of neighboring atoms and their relative distance from a central atom in fcc, bcc, or hcp up to 50th layers. The free-software is able to calculate the structural free energy scale and interatomic bond strengths at different neighboring atomic distances depending on the total number of atoms, cutoff length, and the nature of bond type. The results of our calculation in case of structural free energy scale for the first time perfectly reproduced the expected classical hypothesis of linear dependency of bond energy and melting points. Also the results of bond strengths for different type of metallic elements adequately produced the tendencies reported in literature.

Obviously using the enclosed free-software more reliable values of structural free energy scale can be evaluated separately for each metallic element via considering better set of cutoff length and power of potential energy related to nature of bond types in particular crystal structure.

In next paper the mathematical series and other free-software for estimating the surface bond strength and related surface properties of all metallic elements at different crystal structures and crystal planes will be submitted.

## Figures and Tables

**Figure 8:**
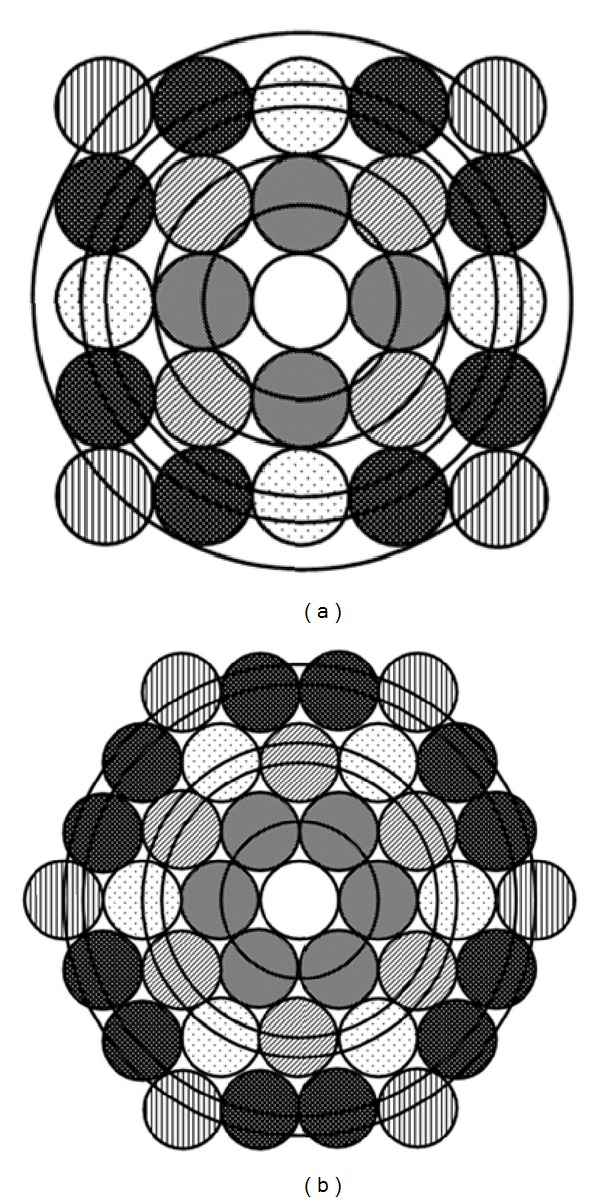
(a) 2D square lattice. (b) 2D hexagonal lattice.

**Table 1:** First 15 numbers of neighboring atoms and their relative distances for each atomic group in fcc, bcc, and hcp crystallography while the relations $a=\sqrt{2}d_{a},\sqrt{3}a=2d_{a}$, and *a* = *d*_*a*_ (*a*: lattice distance) are held in those crystal structure, respectively (knowing that: *g*_crystal_(*i*) = *d*_*i*_/*d*_*a*_).

*i*	ccn(*i*) in fcc	Distance in fcc as (*d*_*i*_/*d*_*a*_)^2^	Distance in fcc as (*d*_*i*_/*a*)^2^	ccn(*i*) in bcc	Distance in bcc as (*d*_*i*_/*d*_*a*_)^2^	Distance in bcc as (*d*_*i*_/*a*)^2^	ccn(*i*) in hcp	Distance in hcp as (*d*_*i*_/*d*_*a*_)^2^	Distance in hcp as (*d*_*i*_/*a*)^2^
1	12	1	4/2	8	3/3	4/3	12	3/3	3/3
2	6	2	4/4	6	3/4	4/4	6	3/6	3/6
3	24	3	4/6	12	3/8	4/8	2	3/8	3/8
4	12	4	4/8	24	3/11	4/11	18	3/9	3/9
5	24	5	4/10	8	3/12	4/12	12	3/11	3/11
6	8	6	4/12	6	3/16	4/16	6	3/12	3/12
7	48	7	4/14	24	3/19	4/19	12	3/15	3/15
8	6	8	4/16	24	3/20	4/20	12	3/17	3/17
9	36	9	4/18	24	3/24	4/24	6	3/18	3/18
10	24	10	4/20	32	3/27	4/27	6	3/19	3/19
11	24	11	4/22	12	3/32	4/32	12	3/20	3/20
12	24	12	4/24	48	3/35	4/35	24	3/21	3/21
13	72	13	4/26	30	3/36	4/36	6	3/22	3/22
14	48	15	4/30	24	3/40	4/40	12	3/25	3/25
15	12	16	4/32	24	3/43	4/43	12	3/27	3/27

**Table 2:** Calculated structural free cohesive energy scale *E* ^av^ of most pure crystalline elements in three main crystallographic structures in [kJ/mol] based on the cohesive energy scale in [1].

fcc*	at *T*_m_**	*E*_0k_^av^	*E*_*T*_*m*__^av^	bcc*	at *T*_m_**	*E*_0k_^av^	*E*_*T*_*m*__^av^	hcp*	at *T*_m_**	*E*_0k_^av^	*E*_*T*_*m*__^av^
Ca	bcc	49.610	55.800	Li	bcc	25.087	22.700	Be	bcc	69.309	78.070
Sr	bcc	51.712	52.540	Na	bcc	20.821	18.560	Mg	hcp	41.618	36.590
Rh	fcc	100.593	86.400	K	bcc	18.977	16.830	Sc	bcc	83.824	90.680
Ir	fcc	122.614	105.09	Rb	bcc	17.721	15.640	Y	bcc	82.411	90.030
Ni	fcc	77.813	66.86	Cs	bcc	17.112	15.090	Ti	bcc	88.974	97.030
Pd	fcc	81.294	70.610	Ba	bcc	58.575	50.040	Zr	bcc	97.193	106.340
Pt	fcc	91.404	79.130	V	bcc	126.739	109.600	Hf	bcc	116.398	125.110
Cu	fcc	59.976	52.540	Nb	bcc	159.683	137.620	Re	hcp	159.975	136.880
Ag	fcc	54.518	47.740	Ta	bcc	188.851	163.050	Ru	hcp	116.029	100.010
Au	fcc	59.255	51.770	Cr	bcc	123.929	106.590	Os	hcp	150.348	130.820
Al	fcc	41.231	36.110	Mo	bcc	169.613	144.930	Co	fcc	81.932	68.410
Pb	fcc	26.422	23.230	W	bcc	216.034	184.160	Tl	bcc	25.929	28.870
In	fcc	18.741	16.610	Fe	bcc	106.917	90.530	Zn	hcp	31.225	27.460
								Cd	hcp	26.757	23.540

fcc *n* = 8, *i* = 11, bcc *n* = 9, *i* = 10, hcp *n* = 9, *i* = 13.

*Crystal structure between 0 K and 298 K [16].

**The crystal structure at which the metal will melt.

**Table 3:** Comparison between our *E*_pair_ (*i*) calculations at 0 K from Table 1 and (4) and (11), with reported estimation bonds strength of first 3 neighboring atoms inside fcc metals [8].

fcc	^ 1^b. t.	ccn(*i*)	[8]	^ 2^Equations (4) and (11)	^3^⋯	fcc	^ 1^b. t.	ccn(*i*)	[8]	^ 2^Equations (4) and (11)	^3^⋯
Au	A or 1	12	31.34491	29.627500	31.51	Pt	1	12	40.31798	45.702000	48.61
	B or 2	6	0.22635	1.851719	0.1230		2	6	0.34635	2.856375	0.1898
	C or 3	24	0.00548	0.365772	0.0048		3	24	0.00959	0.564222	0.0074
	4	12		0.115732			4	12		0.178523	

Ag	1	12	25.88074	27.259000	28.99	Pd	1	12	49.24018	40.647000	43.23
	2	6	0.18539	1.703688	0.1132		2	6	0.43890	2.540438	0.1688
	3	24	0.00446	0.336531	0.0044		3	24	0.01250	0.501815	0.0065
	4	12		0.106480			4	12		0.158777	

Al	1	12	25.50269	20.615500	21.92	Ni	1	12	48.16400	38.906500	41.38
	2	6	0.38510	1.288469	0.0856		2	6	0.64762	2.431656	0.1616
	3	24	0.01643	0.254512	0.0033		3	24	0.02529	0.480327	0.0063
	4	12		0.080529			4	12		0.151979	

Cu	1	12	37.39334	29.988000	31.89						
	2	6	0.45492	1.874250	0.1245						
	3	24	0.01644	0.370222	0.00486						
	4	12		0.117141							

^1^b. t. is bond type.

^2^With *n* = 8  and  *i* = 11.

^3^Equations (4) and (11) with *n* = 16  and  *i* = 3.

**Table 4:** Comparison between our *E*_pair_ (*i*) calculations at 0 K from Table 1 and (4) and (11), with reported estimation bonds strength of first 3 neighboring atoms inside bcc metals [9].

bcc	^ 1^b. t.	ccn(*i*)	[9]	^ 2^Equations (4) and (11)	^3^⋯	bcc	^ 1^b. t.	ccn(*i*)	[9]	^ 2^Equations (4) and (11)	^3^⋯
Ba	A or 1	8	26.94813	29.287500	34.27	Cr	1	8	76.53049	61.964500	72.51
	B or 2	6	2.63391	8.025239	2.573		2	6	15.03984	16.979255	5.444
	C or 3	12		0.354669	0.0050		3	12	0.10841	0.750384	0.0106
	4	24		0.084618			4	24		0.179028	

V	1	8	53.13139	63.369500	74.16	Mo	1	8	91.04682	84.806500	99.25
	2	6	9.70330	17.364247	5.568		2	6	15.64323	23.238325	7.452
	3	12	0.05577	0.767399	0.0010		3	12	0.07440	1.026999	0.0145
	4	24		0.183088			4	24		0.245023	

Nb	1	8	95.48674	79.841500	93.44	W	1	8	107.09495	108.017000	126.41
	2	6	15.15142	21.877837	7.015		2	6	18.22915	29.598370	9.491
	3	12		0.966873	0.0137		3	12	0.08422	1.308076	0.0185
	4	24		0.230679			4	24		0.312083	

Ta	1	8	109.20519	94.425500	110.5	Fe	1	8	59.67579	53.458500	62.56
	2	6	17.31214	25.874084	8.296		2	6	11.84176	14.648476	4.697
	3	12		1.143484	0.0162		3	12	0.08796	0.647377	0.0091
	4	24		0.272815			4	24		0.154453	

^1^b. t. is bond type.

^2^With *n* = 9 and *i* = 13.

^3^Equations (4) and (11) with *n* = 18 and *i* = 3.

**Table 5:** Comparison between our *E*_pair_ (*i*) calculations at 0 K from Table 1 and (4) and (11), with reported estimation bonds strength of first 5 neighboring atoms inside hcp metals [10].

hcp	^ 1^b. t.	ccn(*i*)	^ 2,3^Reference [10]	^ 4^Equations (4) and (11)	^5^⋯	hcp	^ 1^b. t.	ccn(*i*)	^ 2,3^Reference [10]	^ 4^Equations (4) and (11)	^5^⋯
Be	A, B, 1	12	26.63648	34.654500	36.02	Hf	1	12	63.18858	58.199000	60.49
	C or 2	6	0.81081	1.531527	0.0703		2	6	0.2562	2.572057	0.1181
	D or 3	2	0.2014	0.419663	0.0052		3	2	0.02446	0.704784	0.0088
	E, F, 4	18	0.06083	0.247010	0.0018		4	18	0.00410	0.414830	0.0030
	G or 5	12	0.01951	0.100124	0.0003		5	12	0.00059	0.168149	0.0005
	6	6		0.067685			6	6		0.113670	

Mg	1	12	10.26661	20.809000	21.63	Re	1	12	77.23997	79.987500	83.14
	2	6	0.0961	0.919637	0.0422		2	6	0.66184	3.534981	0.1623
	3	2	0.0092	0.251995	0.0031		3	2	0.06507	0.968641	0.0121
	4	18	0.00285	0.148322	0.0010		4	18	0.01845	0.570133	0.0042
	5	12	0.00041	0.060121	0.0001		5	12	0.00272	0.231100	0.0006
	6	6		0.040643			6	6		0.156226	

Sc	1	12	30.80765	41.912000	43.56	Co	1	12	61.57229	40.966000	42.58
	2	6	0.24091	1.852266	0.0850		2	6	0.79776	1.810459	0.0831
	3	2	0.02786	0.507550	0.0063		3	2	0.09046	0.496094	0.0062
	4	18	0.00634	0.298740	0.0022		4	18	0.03042	0.291997	0.0021
	5	12	0.00107	0.121092	0.0003		5	12	0.00504	0.118359	0.0003
	6	6		0.081859			6	6		0.080012	

Y	1	12	33.92099	41.205500	42.83	Zn	1	12	13.52421	15.612500	16.22
	2	6	0.1627	1.821043	0.0836		2	6	0.06636	0.689982	0.0316
	3	2	0.01822	0.498995	0.0062		3	2	0.00438	0.189066	0.0023
	4	18	0.00298	0.293704	0.0021		4	18	0.00241	0.111282	0.0008
	5	12	0.00049	0.119051	0.0003		5	12	0.00008	0.045108	0.0001
	6	6		0.080479			6	6		0.030493	

Ti	1	12	41.89133	44.487000	46.24	Cd	1	12	15.34774	13.378500	13.9
	2	6	0.50957	1.966066	0.0903		2	6	0.03772	0.591252	0.0271
	3	2	0.07548	0.538733	0.0067		3	2	0.00206	0.162012	0.0020
	4	18	0.01884	0.317094	0.0023		4	18	0.00097	0.095359	0.0007
	5	12	0.00393	0.128532	0.0003		5	12	0.00002	0.038653	0.0001
	6	6		0.086889			6	6		0.026130	

Zr	1	12	44.28581	48.596500	50.51						
	2	6	0.37696	2.147682	0.0986						
	3	2	0.04472	0.588499	0.0074						
	4	18	0.01056	0.346385	0.0025						
	5	12	0.00183	0.140405	0.0004						
	6	6		0.094915							

^1^b. t. is bond type.

^2^For type 1 nearest neighbor bonds A or B.

^3^For type 4 nearest neighbor bonds E or F.

^4^With *n* = 9  and  *i* = 13.

^5^Equations (4) and (11) with *n* = 18  and  *i* = 5.

**Table 6:** Value of $E_{\text{inner}}^{\text{total}}$ in [kJ/mol] at 0 K and melting point of some selected metals from [1].

Metal	$E_{\text{inner},T_{m}}^{\text{total}}$	$E_{\text{inner},\text{0k}}^{\text{total}}$	Metal	$E_{\text{inner},T_{m}}^{\text{total}}$	$E_{\text{inner},\text{0k}}^{\text{total}}$	metal	$E_{\text{inner},T_{m}}^{\text{total}}$	$E_{\text{inner},\text{0k}}^{\text{total}}$
Ca	275.99	317.35	Li	112.30	124.08	Be	386.14	432.76
Sr	259.90	330.80	Na	91.83	102.98	Mg	228.47	259.86
Rh	552.72	643.48	K	83.25	93.86	Sc	448.51	523.39
Ir	672.28	784.35	Rb	77.39	87.65	Y	445.30	514.57
Ni	427.72	497.76	Cs	74.64	84.64	Ti	479.95	555.55
Pd	451.73	520.03	Ba	247.52	289.71	Zr	525.99	606.87
Pt	506.19	584.70	V	542.08	626.85	Hf	618.81	726.78
Cu	336.14	383.66	Nb	680.69	789.79	Re	854.70	998.87
Ag	305.45	348.75	Ta	806.44	934.05	Ru	624.50	724.48
Au	331.19	379.05	Cr	527.23	612.95	Os	816.83	938.76
Al	231.05	263.75	Mo	716.83	838.90	Co	437.62	511.58
Pb	148.66	169.02	W	910.89	1068.5	Tl	142.82	161.90
In	106.26	119.89	Fe	447.77	528.81	Zn	171.46	194.97
						Cd	147.03	167.07

Values of inner cohesion energy of other elements can be collected from [1].

## Appendices

### A. Application of Theta Series for Calculating Number of Neighboring Atoms and Their Relative Distance from a Central Atom in a Close Pack Structure

Assuming the origin of coordinates at the center of an arbitrary atom in a close pack structure, theta series are able to calculate number of neighbors and their distance from this central atom. These series are used for 2D (circular) or 3D (spherical) close pack structures. Let *S*_*n*_ denote the number of atoms at distance $\sqrt{n}$ from the origin. These *S*_*n*_ atoms form a shell of radius $\sqrt{n}$. Central atom, its neighbors, and shells are shown in Figures 8(a) and 8(b) for 2D square lattice and 2D hexagonal lattice, respectively.

Theta series for a close packing structure (Λ) is given by

(A.1) $$\begin{array}{c} \sum_{x\in \Lambda }q^{N(x)}=\sum_{n}S_{n}q^{n}. \end{array}$$

This equation is a power series in the variable *q*. *S*_*n*_ is the number of atoms (ccn in (4)), $\sqrt{n}$ is the distance from the origin (*d*_*i*_ in (5)), and *N*(*x*) = *x* · *x* is norm of a vector *x*.

For example in 2D square lattice shown in Figure 8(a), theta series is described by the following equation:

(A.2) $$\begin{array}{c} \theta_{3}{\left(q\right)}^{2}=1+4q+4q^{2}+4q^{4}+8q^{5}+4q^{8}+\cdots . \end{array}$$

This power series predicts that there are 4, 4, 4, 8, 4,… neighbors at distance of $\sqrt{1}$, $\sqrt{2}$, $\sqrt{4}$, $\sqrt{5}$, and $\sqrt{8}$, respectively, from the central atom as shown in Figure 8(a).

Similarly, for hexagonal lattice shown in Figure 8(b), theta series is introduced as follows:

(A.3) θ2(q2)θ2(q6)+θ3(q2)θ3(q6)=1+6q2+6q6+6q8 +12q14+6q18+⋯.

In the same way, all 2D and 3D close pack structures can be expressed using the following three special Jacobi theta series:

(A.4) θ2(q)=∑m=−∞∞q(m+1/2)2=2q1/4(1+q2+q6+⋯+qn(n+1)+⋯),θ3(q)=∑m=−∞∞qm2=1+2q+2q4+2q9+2q16+⋯,θ4(q)=θ3(−q)=1−2q+2q4−2q9+2q16−⋯.

The Jacobi theta series satisfy large number of identities using which they can be simplified.

For some clusters the following series is also helpful:

(A.5) $$\begin{array}{c} \psi \left(q\right)=\sum_{m=-\infty }^{+\infty }q^{{\left(m+1/k\right)}^{2}}, \end{array}$$

where *k* is a nonzero number.

For fcc structure, theta series are given below which can predict up to 10 numbers of neighbors and their distance from central atom:

(A.6) 12θ3(q)3+θ4(q)3=1+12q2+6q4+24q6 +12q8+24q10 +8q12+48q14+6q16 +36q18+24q20+⋯.

Similarly for hcp

(A.7) θ2(q16/3){θ2(q2)ψ6(q6)+θ3(q)3ψ3(q6)} +θ3(q16/3){θ2(q2)θ2(q6)+θ3(q2)θ3(q6)}=1+12q2+6q4+2q16/3+18q6+12q22/3 +6q8+12q10+12q34/3+6q12+6q38/3+⋯

and in bcc

(A.8) θ2(q4)3+θ3(q4)3=1+8q3+6q4+12q8+24q11 +8q12+6q16+24q19+24q20 +24q24+32q27+⋯.

Sloane and Teo in [21] considered the *d*_*i*_ (first coordination distance) by $\sqrt{2}$, $\sqrt{3}$, and $\sqrt{2}$ during the construction of theta series for fcc, bcc, and hcp structure, respectively. Although in this paper we considered a more general algorithm of *d*_*i*_ = 1. Therefore, for transferring the series of distances for fcc, bcc, and hcp in (A.6), (A.8), and (A.7) into our values in Table 1, in above equations one should multiply the theta series values by 2^−1/2^, 3^−1/2^, and 2^−1/2^, respectively. For more information about theta series and magic numbers [21] is useful.

### B. Selected Values of *E*_inner_^total^

For more details see Table 6.
